# Eicosapentaenoic Acid (EPA) Alleviates LPS-Induced Oxidative Stress via the PPARα–NF-κB Axis

**DOI:** 10.1155/omcl/3509596

**Published:** 2025-06-10

**Authors:** Haya AlAbduljader, Halemah AlSaeed, Amenah Alrabeea, Ameenah Sulaiman, Mohammed J. A. Haider, Fahd Al-Mulla, Rasheed Ahmad, Fatema Al-Rashed

**Affiliations:** ^1^Animal and Imaging Core Facility, Dasman Diabetes Institute, Kuwait City, Kuwait; ^2^Immunology and Microbiology Department, Dasman Diabetes Institute, Kuwait City, Kuwait; ^3^Department of Pharmacology and Toxicology, Faculty of Medicine, Kuwait University, Kuwait City, Kuwait; ^4^Department of Biotechnology, American International University, Al-Jahra, Saad Al Alabdullah, Kuwait; ^5^Department of Biological Sciences, Faculty of Science, Kuwait University, P.O. Box 5969, Safat 13060, Kuwait; ^6^Translational Medicine Department at the Dasman Diabetes Institute, Dasman, Kuwait

## Abstract

Metabolic-endotoxemia, characterized by the translocation of lipopolysaccharide (LPS) from Gram-negative bacteria into the bloodstream, is a key contributor to chronic low-grade inflammation associated with obesity and type 2 diabetes. This condition exacerbates metabolic disruptions by activating Toll-like receptor 4 (TLR4) on macrophages, leading to the release of pro-inflammatory cytokines and subsequent insulin resistance. Eicosapentaenoic acid (EPA; C20:5 (n-3)), an omega-3 polyunsaturated fatty acid, has demonstrated anti-inflammatory and antioxidative properties, but its precise mechanisms of action in mitigating LPS-induced stress remain unclear. This study investigates the pathways through which C20:5 (n-3) alleviates LPS-induced oxidative stress and inflammation in macrophages. C20:5 (n-3) pretreatment significantly reduced LPS-induced inflammatory responses, decreasing IL-1β and IL-6 expression and IL-1β secretion, and lowering the percentage of HLA-DR^+^ macrophages. C20:5 (n-3) also attenuated ER stress, evidenced by reduced expression of ATF4, DDIT3, HSPA5/GRP78, BIP, and CHOP at both gene and protein levels. Oxidative stress was mitigated, as shown by decreased HIF1α expression, reduced ROS levels, and preservation of mitochondrial membrane potential. Importantly, C20:5 (n-3) increased the expression of PPARα and FABP5 while inhibiting NF-κB activation independently of the TLR4-IRF5 pathway. The protective effects of C20:5 (n-3) was abolished by PPARα inhibition with GW9662, indicating that C20:5 (n-3)'s action is PPARα-dependent. This study highlights the modulatory role of C20:5 (n-3) in alleviating LPS-induced oxidative stress and inflammation in macrophages through activation of the FABP5/PPARα/NF-κB axis, independently of TLR4-IRF5 signaling. These findings reveal a novel mechanism for C20:5 (n-3)'s anti-inflammatory effects and suggest that targeting the FABP5/PPARα pathway may offer therapeutic potential for treating metabolic disorders associated with chronic inflammation.

## 1. Introduction

Obesity and type 2 diabetes are rapidly emerging as significant global health challenges, frequently co-occurring with other metabolic disorders, including insulin resistance, metabolic syndrome, and cardiovascular disease [1, 2]. A common feature across these conditions is chronic low-grade inflammation, which is partly attributed to increased levels of circulating proinflammatory cytokines. Although the precise triggers of this inflammatory state are not fully understood, the translocation of lipopolysaccharide (LPS) from Gram-negative bacteria into the bloodstream has been suggested as an initiating factor [3]. This process, known as metabolic endotoxemia, is often exacerbated by high-fat diets, which increase plasma LPS levels, promoting inflammation and metabolic disruptions [4]. LPS mainly acts through Toll-like receptor 4 (TLR4) on immune cells (primarily macrophages), where it triggers a cascade of inflammatory mediators such as IL-1β, IL-6, and TNFα. These mediators subsequently drive insulin resistance and metabolic dysfunction [5].

LPS-induced inflammation is known to contribute to endoplasmic reticulum (ER) stress and oxidative stress, both of which are pivotal in the pathogenesis of metabolic diseases. ER stress arises from an accumulation of misfolded proteins in the ER, initiating the unfolded protein response (UPR) to restore cellular homeostasis [6]. Persistent ER stress, however, can lead to cell death through apoptosis. Similarly, oxidative stress, caused by an imbalance between reactive oxygen species (ROS) production and detoxification, damages cellular components, including lipids, proteins, and DNA, and further amplifies inflammatory signaling pathways [7, 8]. Together, the interplay of ER stress, oxidative stress, and inflammation forms a vicious cycle that underpins the progression of obesity and type 2 diabetes.

Omega-3 polyunsaturated fatty acids (PUFAs), especially eicosapentaenoic acid (EPA; C20:5 (n-3)), have demonstrated protective effects against inflammation and oxidative stress [9]. C20:5 (n-3), a 20-carbon chain with five double bonds, is a long-chain omega-3 fatty acid primarily found in fish oil. It enhances lipid and carbohydrate metabolism and exerts anti-inflammatory effects by interacting with several nuclear receptors. C20:5 (n-3) directly modulates inflammation by engaging with peroxisome proliferator-activated receptors (PPARs), which regulate genes involved in lipid metabolism, energy balance, and inflammation [10].

Although fatty acids like C20:5 (n-3) are recognized for their anti-inflammatory properties, the underlying mechanistic pathways remain poorly understood, particularly in the context of LPS-induced endotoxemia. In the present study, we aimed to investigate the mechanistic pathway through which C20:5 (n-3) alleviates LPS-induced oxidative stress, with a focus on the PPARα–NF-κB axis. Using a cell culture model, we examined C20:5 (n-3)'s effects on inflammation, membrane potential, and ROS levels. Our findings indicate that EPA's anti-inflammatory action requires the activation of PPARα and FABP5 to inhibit NF-κB and is independent of the TLR4-IRF5 pathway. This study provides insights into the potential therapeutic role of C20:5 (n-3) in modulating cellular stress responses associated with obesity and metabolic diseases.

## 2. Materials and Methods

### 2.1. Cell Culture

THP-1 human monocytic cells, obtained from the American Type Culture Collection (ATCC), Manassas, VA, USA, were cultured in RPMI-1640 medium (Gibco, Thermo Fisher Scientific, Waltham, MA, USA). The medium was enriched with 10% fetal bovine serum (Gibco, Thermo Fisher Scientific, Waltham, MA, USA), 2 mM L-glutamine (Gibco, Thermo Fisher Scientific, Waltham, MA, USA), 1 mM sodium pyruvate (Gibco, Thermo Fisher Scientific, Waltham, MA, USA), 10 mM HEPES (Gibco, Thermo Fisher Scientific, Waltham, MA, USA), 100 µg/mL Normocin (InvivoGen, San Diego, CA, USA), and antibiotics comprising 50 U/mL penicillin and 50 µg/mL streptomycin (Gibco, Thermo Fisher Scientific, Waltham, MA, USA). The cells were maintained at 37°C in a humidified atmosphere containing 5% CO_2_.

### 2.2. Macrophage Differentiation

Before stimulation, THP-1 monocytes were differentiated into macrophages as published previously by AlSaeed et al. [11]. Briefly, cells were exposed to 10 ng/mL phorbol-12-myristate-13-acetate (PMA, Sigma–Aldrich, Merck KGaA, Darmstadt, Germany) for 3 days. This was followed by an additional 3-day resting period in RPMI medium devoid of serum and PMA to ensure the cells were adequately primed for further experiments.

### 2.3. Cell Stimulation

Monocytes were seeded into 12-well plates (Costar, Corning Incorporated, Corning, NY, USA) at a density of 1 × 10^6^ cells per well, unless noted otherwise. Differentiation into macrophages was done followed the previously outlined protocol of AlSaeed et al. [11]. The cells were then pre-incubated for 24 h at 37°C with either 200 µM C20:5 (n-3) (cis-5,8,11,14,17-Eicosapentaenoic acid (EPA); Cat # E2011, Sigma–Aldrich, Merck KGaA, Darmstadt, Germany) or 24% bovine serum albumin (BSA; Sigma–Aldrich, Merck KGaA, Darmstadt, Germany) which served as the vehicle control for C20:5 (n-3). Posttreatment, the cells were exposed overnight to 10 ng/mL LPS (10 ng/mL; L4391, Sigma–Aldrich, Merck KGaA, Darmstadt, Germany), which was reconstituted in sterile RPMI-1640 medium. To investigate PPARα involvement, cells were pretreated for 1 h with 32 nM GW9662 (Bio-Techne, Tocris Bioscience, Bristol, UK), dissolved in DMSO (Sigma–Aldrich, Merck KGaA, Darmstadt, Germany). An equivalent volume of 0.01% DMSO was used as vehicle control in these experiments. Following GW9662 pretreatment, cells were treated with C20:5 (n-3) or vehicle (24% BSA) for 24 h and then challenged overnight with LPS.

### 2.4. Preparation of BSA-Fatty Acid Complexes

Complexes of BSA (Sigma–Aldrich, Merck KGaA, Darmstadt, Germany) and fatty acids were prepared using a method adapted from van Harken, Dixon, and Heimberg [12, 13]. Briefly, a 24% (w/v) BSA (Sigma–Aldrich, Merck KGaA, Darmstadt, Germany) solution was prepared by gradually dissolving fatty-acid-free BSA (Cat # A6003, Sigma–Aldrich, Merck KGaA, Darmstadt, Germany) in 150 mM NaCl (Sigma–Aldrich, Merck KGaA, Darmstadt, Germany), adjusting the final pH to 7.4 with 5M sodium hydroxide (NaOH; Sigma–Aldrich, Merck KGaA, Darmstadt, Germany). The solution was then filtered through a 0.22 μm filter (Millipore, Merck KGaA, Darmstadt, Germany) under sterile conditions and aliquoted for storage at –20°C until use. To prepare the fatty acid/BSA solution (Sigma–Aldrich, Merck KGaA, Darmstadt, Germany), C20:5 (n-3) (cis-5,8,11,14,17-eicosapentaenoic acid (EPA); Cat # E2011, Sigma–Aldrich, Merck KGaA, Darmstadt, Germany) was added to the 24% BSA (Sigma–Aldrich, Merck KGaA, Darmstadt, Germany) solution to a final concentration of 10 mM. This mixture was gently heated and stirred on a hot plate until an emulsion formed. The emulsion was then stored at –20°C until needed.

### 2.5. Flow Cytometry Analysis

#### 2.5.1. Staining of Cell-Surface Markers

Monocytic cells were seeded in 24-well plates at 0.5 x 10^6^ cells/well. Cells were transformed into macrophages and pretreated as previously described. To detach macrophages, cells were harvested by gentle pipetting in ice-cold phosphate-buffered saline (PBS) 1x PBS (Sigma–Aldrich, Merck KGaA, Darmstadt, Germany) supplemented with 0.5 mM disodium ethylenediaminetetraacetic acid (EDTA; Sigma–Aldrich, Merck KGaA, Darmstadt, Germany). Harvested cells were then resuspended in FACS staining buffer that was prepared in-house using PBS (1x; Gibco, Thermo Fisher Scientific, Waltham, MA, USA) supplemented with 1% BSA (Sigma–Aldrich, Merck KGaA, Darmstadt, Germany), 2 mM disodium EDTA (Sigma–Aldrich, Merck KGaA, Darmstadt, Germany), and 0.05% sodium azide (Sigma–Aldrich, Merck KGaA, Darmstadt, Germany). The solution was filter-sterilized using a 0.22 μm membrane filter (Millipore, Merck KGaA, Darmstadt, Germany) and stored at 4°C until use. Cells were blocked with 20 µg human IgG (Sigma–Aldrich, Merck KGaA, Darmstadt, Germany) for 30 min on ice, followed by three washes and resuspended in 100 µL of FACS buffer and incubated with anti-CD11b PE-Cy7 (Cat # 557743; BD Pharmingen, San Jose, CA, USA) and anti-HLA-DR PerCP-Cy5.5 (Cat # 560652; BD Pharmingen, San Jose, CA, USA) or appropriate isotype control antibodies (Cat # 558055; BD Phosflow, Cat # 559529; BD Phosflow, Cat # 560542; BD Pharmingen, or Cat # 560817; BD Phosflow, San Jose, CA, USA) on ice for 30 min.

Cells were washed three times with FACS buffer to remove nonspecific binding, then resuspended in FACS buffer for analysis on a FACSCanto (BD Biosciences, San Jose, CA, USA) flow cytometer. Data analysis was performed using BD FACSDiva Software 8 (BD Biosciences, San Jose, CA, USA) or FlowJo software (FlowJo, Ashland, OR, USA). Unstained cells were used to define gating, and median fluorescence intensity (MFI) was measured. The gating strategy is summarized in the Supporting Information 1: Figure S1A.

#### 2.5.2. Staining of Intracellular Markers

For intracellular staining, cells were incubated with fixation/permeabilization buffer (Cat # 00-5523-00, eBioscience, Thermo Fisher Scientific, San Diego, CA, USA) for 20 min at 4°C, followed by washing with Perm Wash Buffer (BD Biosciences, San Jose, CA, USA). Cells were then stained with antihuman IRF5 Alexa Fluor 488 (Cat # IC4508G; R&D Systems, Minneapolis, MN, USA) and anti-NF-κB p65 (pS529)-PE (Cat # 558423; BD Biosciences, San Jose, CA, USA) or mouse anti-human HIF1α Alexa Fluor 647 (Cat # 565924; BD Biosciences, San Jose, CA, USA) at the recommended concentrations according to the manufacturer's instructions.

Samples were acquired on a BD FACSCanto (BD Biosciences, San Jose, CA, USA) flow cytometer, and data analysis was performed using BD FACSDiva Software 8 (BD Biosciences, San Jose, CA, USA) or FlowJo software (FlowJo, Ashland, OR, USA). Unstained cells were used to define gating, and MFI was measured. The gating strategy is summarized in Supporting Information 1: Figure S1B.

#### 2.5.3. Analysis of Intracellular Reactive Oxygen Species by Flow Cytometry

To assess intracellular ROS levels, we used the cell-permeable fluorogenic probe 2′, 7′-dichlorodihydrofluorescein diacetate (DCFH-DA/KP06003; Bioquochem, Asturias, Spain). This probe enters cells, and it is converted by intracellular not specific esterases into highly fluorescent 2′, 7′-dichlorofluorescein (DCF) upon reaction with ROS, serving as an indicator of intracellular oxidative stress. The assay was performed following the manufacturer's instructions. Briefly, THP-1 transformed macrophages were preincubated with 10 µM DCFH-DA (Bioquochem, Asturias, Spain) at 37°C for 30 min. Cultures were then pretreated with 200 µM C20:5 (n-3) (cis-5,8,11,14,17-eicosapentaenoic acid (EPA); Cat # E2011, Sigma–Aldrich, Merck KGaA, Darmstadt, Germany) and/or 10 μM GW9662 (PPARα antagonist, Bio-Techne, Tocris Bioscience, Bristol, UK) or 50 μM tert-butyl hydroperoxide (TBHP; Sigma–Aldrich, Merck KGaA, Darmstadt, Germany) solution serving as a positive control, followed by 10 ng/mL LPS from *E. coli* O111:B4 (LPS; Cat # L4391, Sigma–Aldrich, Merck KGaA, Darmstadt, Germany) stimulation. After 4 h of incubation, cells were harvested, rinsed three times with PBS 1x PBS (Gibco, Thermo Fisher Scientific, Waltham, MA, USA). DCF fluorescence was measured by flow cytometry using excitation/emission wavelengths of 488 nm/525 nm, respectively. For each sample, 10,000 events were recorded using the BD FACSCanto (BD Biosciences, San Jose, CA, USA). Data analysis was conducted to quantify ROS levels as a measure of oxidative stress in different treatment groups.

See Supporting Information 2: Figure S2 for comparative analysis of vehicle controls

#### 2.5.4. Measurement of Mitochondrial Membrane Potential With JC-1

Changes in mitochondrial membrane potential were assessed using 5,5′, 6,6′-tetrachloro-1,1′, 3,3′-tetraethyl-benzimidazolcarbocyanine iodide (JC-1; Cat # T3168, Thermo Fisher Scientific, Waltham, MA, USA). JC-1 emits either green or red fluorescence depending on the mitochondrial membrane potential: green fluorescence (monomers) indicates depolarized mitochondria (low membrane potential), while red fluorescence (aggregates) reflects polarized mitochondria (high membrane potential) [14]. In this context, the term “polarized” refers to the maintenance of a high electrochemical gradient across the inner mitochondrial membrane, where the matrix side remains negatively charged relative to the intermembrane space, a condition essential for ATP production. This gradient typically ranges between −150 and −180 mV. In contrast, “depolarized” mitochondria exhibit a loss or reduction of this membrane potential, reflecting impaired mitochondrial function. This terminology is well established and widely accepted in mitochondrial biology and is commonly used in JC-1-based assays to distinguish between healthy and dysfunctional mitochondria.THP-1 transformed macrophages were pretreated with 200 µM C20:5 (n-3) (cis-5,8,11,14,17-eicosapentaenoic acid (EPA); Cat # E2011, Sigma–Aldrich, Merck KGaA, Darmstadt, Germany) and/or 10 μM GW9662 (PPARα antagonist, Bio-Techne, Tocris Bioscience, Bristol, UK) followed by stimulation with 10 ng/mL LPS from *E. coli* O111:B4 (LPS; Cat # L4391, Sigma–Aldrich, Merck KGaA, Darmstadt, Germany) as per experimental design. After 24 h of incubation, cells were harvested by ice cold 1x PBS (Gibco, Thermo Fisher Scientific, Waltham, MA, USA) supplemented with 0.5 mM EDTA (Sigma–Aldrich, Merck KGaA, Darmstadt, Germany). Cells were centrifugated at 300x g for 5 min and washed twice with prewarmed 1x PBS (Gibco, Thermo Fisher Scientific, Waltham, MA, USA). Cells were then resuspended in 2 µM JC-1 (Thermo Fisher Scientific, Waltham, MA, USA) and incubated at 37°C for 20 min in the dark. Following incubation, cells were washed twice with 1x PBS (Gibco, Thermo Fisher Scientific, Waltham, MA, USA) to remove excess dye and resuspended in 500 mL 1x PBS (Gibco, Thermo Fisher Scientific, Waltham, MA, USA) for analysis. JC-1 fluorescence was measured using a FACSCanto II flow cytometer (BD Biosciences, San Jose, CA, USA) with laser excitation at 488 nm. Emissions were detected at two wavelengths: green fluorescence at 530/30 nm for JC-1 monomers (depolarized mitochondria) and red fluorescence at 585/42 nm for JC-1 aggregates (polarized mitochondria). For each sample, at least 10,000 events were recorded. Data were analyzed using FlowJo software (FlowJo, Ashland, OR, USA). The mitochondrial membrane potential was expressed as the ratio of red (aggregates) to green (monomers) fluorescence intensity, where a higher ratio reflects preserved mitochondrial membrane potential, and a lower ratio indicates mitochondrial depolarization.

See Supporting Information 2: Figure S2 for comparative analysis of vehicle controls.

### 2.6. Quantitative Real-Time Polymerase Chain Reaction

Total RNA was isolated using the RNeasy Mini Kit (Qiagen, Valencia, CA, USA) following the manufacturer's protocol. The quality and quantity of the isolated RNA were assessed using a NanoDrop spectrophotometer (Applied Biosystems, Thermo Fisher Scientific, Waltham, MA, USA) by measuring the absorbance ratios at 260/280 and 260/230 nm. A 260/280 ratio of ~ 2.0 was considered indicative of high RNA purity, and a 260/230 ratio above 1.8 reflected low levels of contaminants such as phenol or guanidine. Only samples meeting these quality criteria were used for downstream applications. Complementary DNA (cDNA) was synthesized from 1 μg of RNA using the High-Capacity cDNA Reverse Transcription Kit Applied Biosystems, Thermo Fisher Scientific, Waltham, MA, USA). Quantitative real-time polymerase chain reaction (qRT-PCR) was carried out on the 7500 Fast Real-Time PCR System Applied Biosystems, Thermo Fisher Scientific, Waltham, MA, USA) using the TaqMan Gene Expression Master Mix Applied Biosystems, Thermo Fisher Scientific, Waltham, MA, USA). Each reaction utilized 1000 ng of cDNA and TaqMan Gene Expression Assay reagents (Applied Biosystems, Thermo Fisher Scientific, Waltham, MA, USA), as detailed in Table 1. Threshold cycle (Ct) values were normalized to GAPDH, and the *∆∆*Ct method was employed to calculate relative mRNA levels compared to the control. Relative gene expression was presented as fold change, with the control set to 1. Data are shown as mean ± SEM, and statistical analysis was conducted with significance determined at *p* < 0.05.

### 2.7. Western Blot Analysis of Protein Expression

Treated cell cultures were lysed using RIPA buffer (Thermo Fisher Scientific, Waltham, MA, USA)) supplemented with a protease and phosphatase inhibitor cocktail (Thermo Fisher Scientific, Waltham, MA, USA) to safeguard against protein degradation. The lysates were centrifuged at 14,000 x g for 15 min at 4°C to eliminate debris. Protein concentration was determined using the Bradford assay (Bio-Rad Laboratories, Hercules, CA, USA), and equal amounts of protein (12.5 µg per sample) were resolved on a 10% SDS-PAGE gel. Gels were run using pre-made 10 × running buffer (Bio-Rad Laboratories, Hercules, CA, USA), diluted to 1 × in double-distilled water (ddH_2_O).

Proteins were transferred onto PVDF membranes (MilliporeSigma, Burlington, MA, USA) using a wet transfer system with 10× Tris-Glycine transfer buffer (Bio-Rad Laboratories, Hercules, CA, USA), also diluted to 1× in ddH_2_O, using the Mini Trans-Blot Cell (Bio-Rad). Membranes were blocked for 1 h at room temperature using either Blotto A or Blotto B (ChemCruz, Santa Cruz Biotechnology, Dallas, TX, USA), depending on the target Blotto A was used for total protein detection, while Blotto B was used for phosphoprotein targets.

After blocking, membranes were incubated overnight at 4°C with primary antibodies specific to the proteins of interest (Table 2). β-Actin was used as a loading control. The following day, membranes were washed with Tris-buffered saline (TBST) (with 0.1% Tween-20; Sigma–Aldrich, Merck KGaA, Darmstadt, Germany) and incubated with HRP-conjugated secondary antibodies (Cell Signaling Technology, Danvers, MA, USA) for 1 h at room temperature. Protein bands were visualized using an ECL detection reagent (Thermo Fisher Scientific, Waltham, MA, USA) and imaged using a ChemiDoc Imaging System (Bio-Rad Laboratories, Hercules, CA, USA). Densitometric analysis was performed using ImageJ software (National Institutes of Health, Bethesda, MD, USA), and protein expression levels were normalized to β-Actin. Full immunoblot images are provided in the supporting PowerPoint file (Supporting Information 1: WB Immunoblots).

### 2.8. Measurement of AP-1/NF-κB Activity

THP-1 XBlue monocytes (InvivoGen, San Diego, CA, USA) were used in this study. These cells are stably transfected with a reporter construct encoding secreted embryonic alkaline phosphatase (SEAP) under the control of a promoter responsive to AP-1 and NF-κB transcription factors. Upon NF-κB activation, SEAP is secreted into the culture supernatant, allowing quantitative assessment of pathway activation. Cells were pretreated following the protocol described by Haider et al. [15]. Following stimulation, SEAP levels were measured in the supernatants after 3 4 h of incubation with Quanti-Blue solution (InvivoGen, San Diego, CA, USA) at room temperature. Absorbance was read at 650 nm using a microplate reader (BioTek Instruments, Winooski, VT, USA).

### 2.9. Sandwich Enzyme-Linked Immunosorbent Assay

Secreted IL-1β protein concentrations were quantified in the media of THP-1-treated cell cultures using sandwich enzyme-linked immunosorbent assay (ELISA) in accordance with the manufacturer's instructions (R&D systems, Minneapolis, MN, USA, Cat # DY279) and as previously published Al-Rashed et al. [16].

### 2.10. Statistical Analysis

Statistical analysis was performed using GraphPad Prism software (La Jolla, CA, USA). Data are shown as the mean ± (SEM), unless otherwise indicated. Parametric data were analyzed by one-way ANOVA followed by Tukey's post hoc multiple comparisons test. For all analyses, data from a minimum of three replicates were used for statistical calculation. A *p* value of 0.05 was considered statistically significant. ns = nonsignificant, *⁣*^*∗*^*p* < 0.05, *⁣*^*∗∗*^*p* < 0.01, *⁣*^*∗∗∗*^*p* < 0.001, and *⁣*^*∗∗∗∗*^*p* < 0.0001.

## 3. Results

### 3.1. C20: 5 (n-3) Downregulates LPS-Induced Inflammatory Response and ER Stress

The anti-inflammatory properties of omega-3 fatty acids have been well documented in numerous studies. However, despite the variety of omega-3 family members, their specific effects on inflammatory outcomes remain partially unclear, with some demonstrating more potent effects in mitigating inflammation than others. One proposed mechanism is that these fatty acids alleviate ER stress, thereby reducing the inflammatory response.

**Figure 1:**
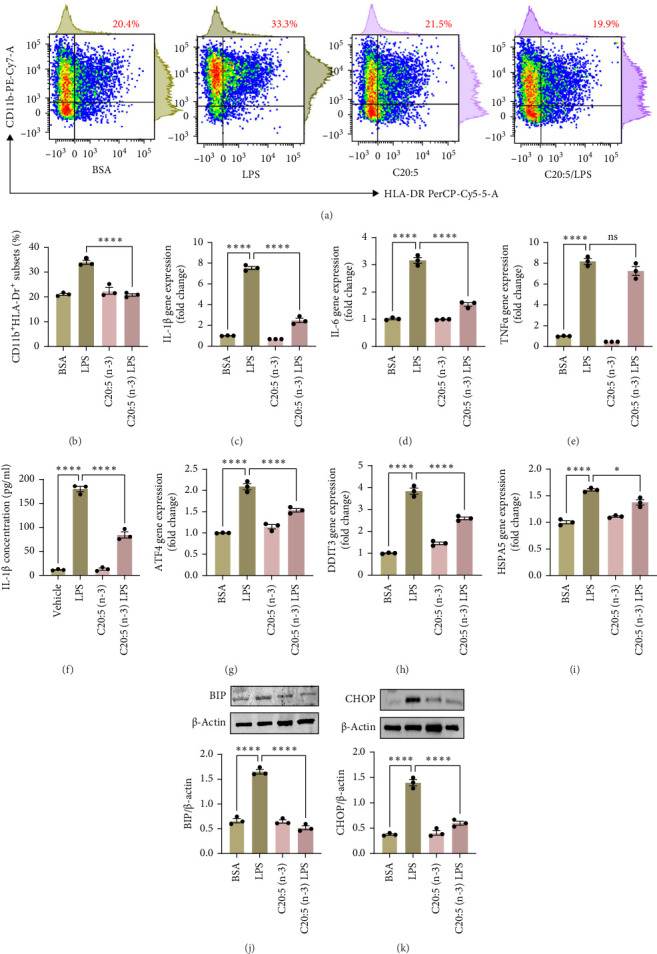
Eicosapentaenoic Acid (EPA; C20:5 (n-3)) reduces LPS-induced inflammatory response, ER stress, and oxidative stress in THP-1 macrophages derived. THP-1 macrophages were pretreated with 200 µM C20:5 (n-3) and subsequently stimulated with LPS (10 ng/mL). (A) Representative dot plots of flow cytometry analysis of HLA-DR + macrophage subsets. (B) Bar graph analysis of HLA-DR + subsets percentage. Quantitative gene expression analysis of (C) IL-1β, (D) IL-6, and (E) TNF-α in THP-1 macrophages. (F) Protein secretion profile of IL-1β in the culture supernatant. Quantitative gene expression analysis of ER stress markers (G) ATF4, (H) DDIT3, and (I) HSPA5 (GRP78) in response to C20:5 (n-3) treatment. Western blot analysis of (J) BIP and (K) CHOP protein levels corrected to β-actin. Data are presented as mean ± SEM, with a minimum of *n* = 3. Statistical significance was assessed using one-way ANOVA followed by Tukey's post-hoc test. *⁣*^*∗*^*p* < 0.05, and *⁣*^*∗∗∗∗*^*p* < 0.0001.

To test this hypothesis and further understand the underlying mechanisms, we utilized a THP-1-derived macrophage model. The cells were pre-incubated with 200 µM of C20:5 (n-3) and subsequently stimulated with 10 ng/mL of LPS. As expected, pretreatment with EPA significantly reduced the percentage of HLA-DR^+^ macrophage subsets compared to cells exposed only to LPS (Figure 1A,B) verifying this anti-inflammatory property.

This effect was also observed at the gene level, where C20:5 (n-3) pretreatment significantly downregulated the gene expression of proinflammatory cytokines IL-1β and IL-6 (Figure 1C,D). Although a reduction in TNF-α gene expression was observed, it did not reach statistical significance (Figure 1E). Furthermore, we tested the secretion profile of these proinflammatory cytokines in the media. As expected, a significant reduction was seen in IL-1β in cells that were pretreated with C20:5 (n-3) (Figure 1F). These results indicate that C20:5 (n-3) effectively mitigates LPS-induced inflammatory responses.

Previous studies have demonstrated that LPS induces ER stress [17]. In our model, C20:5 (n-3) pretreatment significantly reduced the expression of key ER stress markers, including ATF4, DDIT3, and HPAS5/GRP78 at the gene level (Figure 1G–I). This reduction in ER stress was further confirmed at the protein level, as evidenced by decreased expression of BIP, a chaperone associated with HPAS5, and CHOP, a protein marker for DDIT3 (Figure 1J,K). Altogether, these findings suggest that C20:5 (n-3) attenuates both the inflammatory response and ER stress induced by LPS.

### 3.2. C20:5 (n-3) Downregulates LPS-Induced Oxidative Stress

**Figure 2:**
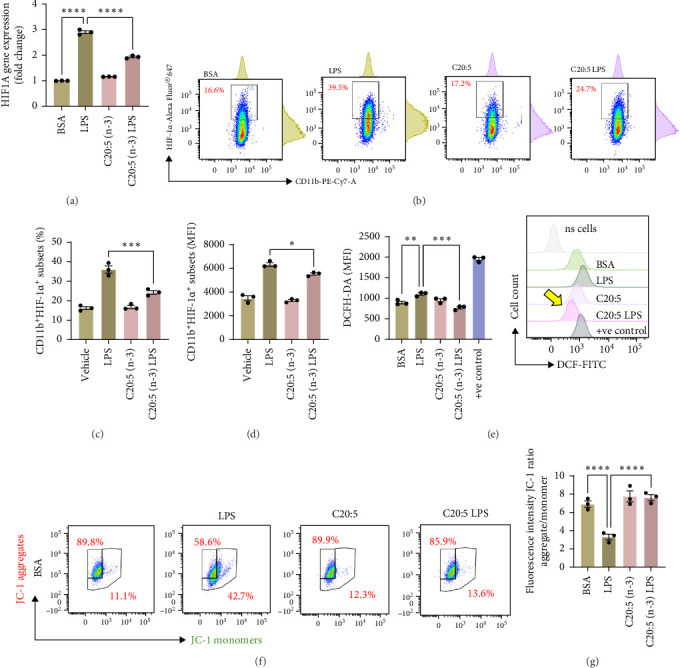
Eicosapentaenoic acid C20:5 (n-3) preserves mitochondrial membrane potential and reduces oxidative stress in LPS-stimulated THP-1-derived macrophages. (A) Quantitative gene expression analysis of HIF1A. (B) Representative dot plots from flow cytometry showing the percentage of CD11b^+^HIF1α^+^ macrophages. (C) Bar graph quantifying the percentage of CD11b^+^HIF1α^+^ cells across treatment groups. (D) Median fluorescence intensity (MFI) of intracellular HIF1α expression in CD11b^+^HIF1α^+^ subsets. (E) Intracellular ROS was measured by flow cytometry (DCFH-DA) with data presented as a bar graph of median fluorescence intensity (MFI) with a representative histogram. (F) Representative dot plots from flow cytometry for JC-1 aggregates (representing polarized mitochondria) and monomers (representing depolarized mitochondria). (G) Bar graphs show an aggregate-to-monomer ratio. Data are presented as mean ± SEM, with a minimum of *n* = 3. Statistical significance was determined using one-way ANOVA followed by Tukey's post hoc test. *⁣*^*∗∗*^*p* < 0.01, *⁣*^*∗∗∗*^*p* < 0.001, and *⁣*^*∗∗∗∗*^*p* < 0.0001.

To further explore the mechanisms by which C20:5 (n-3) mitigates cellular stress, we examined its impact on oxidative stress, a well-known contributor to ER stress and inflammation. Given the role of HIF1α in promoting oxidative stress during inflammatory conditions, we first assessed its gene expression, suggesting that C20:5 (n-3) might mitigate oxidative stress through modulation of this pathway. As expected, LPS stimulation showed significant upregulation in HIF1A gene expression, indicating an induction of oxidative stress similar to our previous observations where pretreatment with C20:5 (n-3) prior to LPS stimulation significantly reduced this upregulation (Figure 2A). To complement the gene expression findings, we additionally evaluated HIF1α protein levels by flow cytometry. Macrophages were gated on CD11b^+^ subsets, and intracellular HIF1α expression was measured. The dot plot (Figure 2B) and bar graph (Figure 2C) show a notable increase in the percentage of CD11b^+^HIF1α^+^ cells upon LPS treatment, which was markedly reduced by C20:5 (n-3) pretreatment. Furthermore, quantification of HIF1α MFI within CD11b^+^HIF1α^+^ cells (Figure 2D) revealed a significant reduction in HIF1α protein expression following C20:5 (n-3) exposure, corroborating our transcript-level findings. To further verify this observation, we conducted a functional analysis for ROS production using a flow cytometric analysis of DCFH-DA. Consistent with a reduction in HIF1α expression, ROS levels were markedly reduced following C20:5 (n-3) treatment (Figure 2E). This indicates that C20:5 (n-3) effectively alleviates oxidative stress, potentially through HIF1α downregulation and related pathways. Furthermore, since mitochondrial dysfunction is a major source of ROS production, we further examined the effect of C20:5 (n-3) on mitochondrial membrane potential using a JC-1 assay. Compared with the LPS-treated group, pretreatment with C20 : 5 (n-3) preserved mitochondrial membrane potential as indicated by a stable aggregate to monomers ratio (Figure 2F,G), suggesting that C20:5 (n-3) prevents mitochondrial depolarization under LPS-induced stress conditions. Collectively, the observed data underscore the multifaceted protective role of C20:5 (n-3) in cellular stress responses, likely mediated through its impact on mitochondrial integrity and oxidative stress regulation.

### 3.3. C20:5 (n-3) Modulates FABP5/PPARα/NF-κβ Signaling Independently of the TLR4-IRF5 Pathway

**Figure 3:**
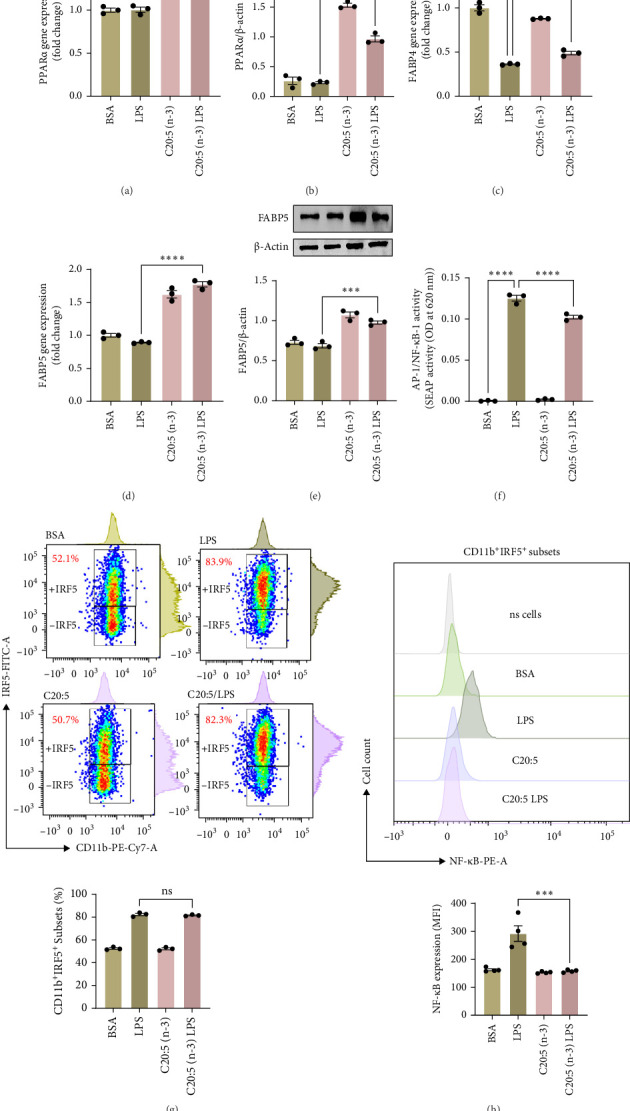
C20:5 (n-3) modulates the PPARα/FABP5/NF-κB pathway independently of IRF5 activation. Quantitative analysis of PPARα expression at the (A) mRNA and (B) protein levels. (C) Quantitative analysis of FABP4 gene expression. Quantitative analysis of FABP5 expression at the (D) mRNA and (E) protein levels. (F) NF-κB reporter monocytic cells SEAP reporter activity. (G) Representative dot plots show the percentage of CD11b+IRF5^+^ subsets and bar graph showing the percentage. (H) Representative flow cytometry histogram and bar graph depicting the median fluorescence intensity (MFI) of NF-κB in CD11b^+^IRF5^+^ subsets. Data are presented as mean ± SEM, with a minimum of *n* = 3. Statistical significance was determined using one-way ANOVA followed by Tukey's post hoc test. *⁣*^*∗∗*^*p* < 0.01, *⁣*^*∗∗∗*^*p* < 0.001 and *⁣*^*∗∗∗∗*^*p* < 0.0001.

To further elucidate the molecular mechanisms underlying the protective effects of C20:5 (n-3), we focused on key modulators involved in lipid metabolism and inflammation. Specifically, we investigated the expression of PPARα, IRF5, and FABP4/5 given their role in mitochondrial function, oxidative stress, and inflammation. PPARα, a nuclear receptor that regulates mitochondrial fatty acid oxidation and energy homeostasis, was significantly reduced by LPS treatment. Interestingly, C20:5 (n-3) elevated PPARα expression at both gene and protein levels (Figure 3A,B, respectively). This observation suggests a key role in re-establishing mitochondrial function and metabolic homeostasis in the presence of inflammatory stimuli such as LPS. Fatty acids (FA), including C20:5 (n-3), are known ligands for PPARs. Their subsequent binding can impact their activity. During this process, FA requires the initial interaction with fatty acid-binding proteins (FABP), which act as chaperones to transport ligands to PPARs. While no significant effect was observed on FABP4 expression when compared to LPS-treated cells, C20:5 (n-3) induced a marked increase in FABP5 at both gene and protein levels (Figure 3C–E). This observation indicates that C20:5 (n-3) might exert its protective effects through this specific pathway, facilitating its interaction with PPARα to promote fatty acid oxidation and anti-inflammatory responses. Given that IRF5 is a key transcription factor activated downstream of Toll-like receptor 4 (TLR4) signaling, we explored its role in modulating the inflammatory response through the activation of the NF-κβ pathway. At the gene level, the expression of IRF5 was abolished under C20:5 (n-3) pretreatment (Figure 3F). Subsequently, C20:5 (n-3) pretreatment significantly reduced NF-κβ activation as demonstrated in THP-1 -NF-κβ reporter cells, where we observed a notable reduction in NF-κβ expression (Figure 3G). To further understand this dynamic and to validate the inhibitory effect of C20:5 (n-3) on the IRF5/NF-κβ pathway, we conducted flow cytometric analysis. Interestingly, while there was no significant change in the percentage of IRF5^+^ subsets, we observed a significant reduction in NF-κβ MFI within these cells, indicating that C20:5 (n-3)'s primary mode of action may not directly inhibit IRF5 expression but instead suppress NF-κβ activation downstream of the TLR4-IRF5 axis (Figure 3H). Together, these results highlight the capacity of C20:5 (n-3) to modulate NFκβ through the FABP5/PPARα/NF-κβ axis independently of TLR4/IRF5.

### 3.4. PPARα Inhibition Abrogates C20:5 (n-3)'s Effect on LPS Stress and Inflammation

**Figure 4:**
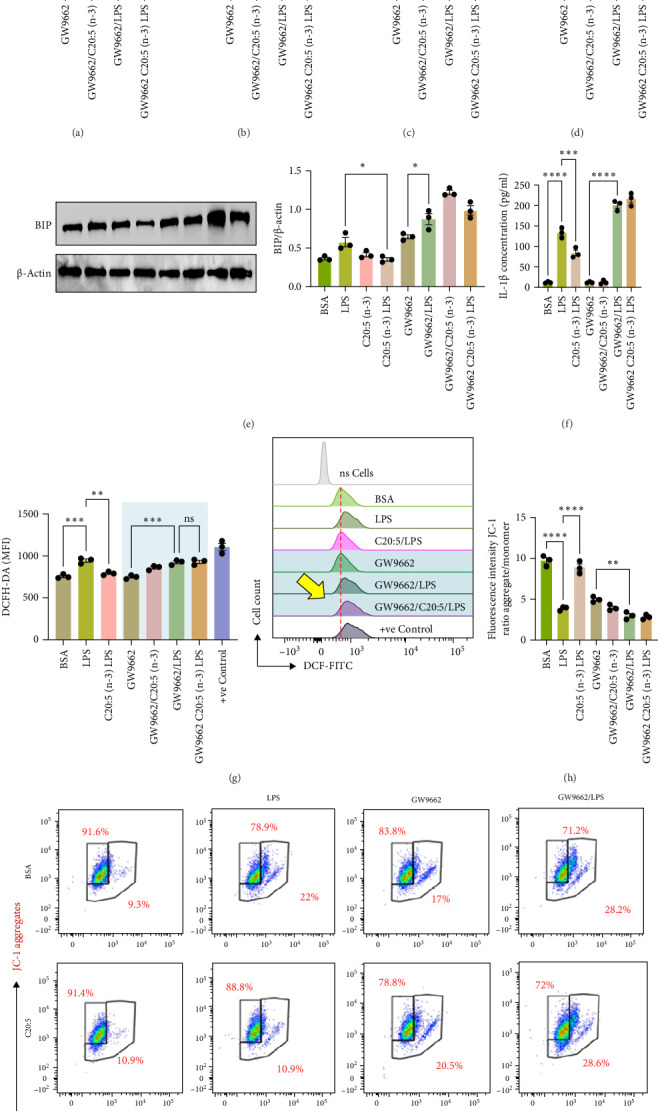
PPARα antagonism abrogates C20:5 (n-3)'s protective effects on LPS-induced inflammatory and cellular stress responses in THP-1 derived macrophages. Cells were pretreated with 200 µM C20:5 (n-3), GW9662 (a PPARα antagonist), or both, followed by LPS (10 ng/mL) stimulation. Quantitative analysis of gene expression for ER stress markers (A) HIF1A, (B) DDIT3, (C) ATF4, and (D) HSPA5. (E) Western blot analysis of BIP protein level. (F) Protein secretion analysis of IL-1β. (G) Bar graph and representative histogram of ROS production measured by DCFH-DA assay. Yellow arrow highlights the histogram corresponding to C20:5 (n-3) pretreated cells co-challenged with LPS. (H) Quantification of mitochondrial membrane potential using a JC-1 assay. (I) Flow cytometric dot plots for JC-1 aggregates and monomers showing mitochondrial polarization status across different treatment groups. Data are presented as mean ± SEM, with a minimum of *n* = 3. Statistical significance was determined using one-way ANOVA followed by Tukey's post hoc test. Significance levels are indicated as follows: *⁣*^*∗*^*p* < 0.05, *⁣*^*∗∗*^*p* < 0.01, *⁣*^*∗∗∗*^*p* < 0.001, *⁣*^*∗∗∗*^*p* < 0.0001; ns = not significant.

To interrogate the mechanistic role of PPARα in mediating the protective effects of the omega-3 fatty acid C20:5 (n-3), we pretreated macrophages with GW9662, a pharmacological PPARα antagonist, prior to C20:5 (n-3) exposure and subsequent LPS stimulation. Under PPARα inhibition, it became evident that C20:5 (n-3)'s ability to mitigate oxidative stress and subsequent ER stress was significantly diminished, as observed in the expression of HIF1A (Figure 4A). This loss of function was also noted in ER stress genes, including DDIT3, ATF4, and HSPA5, which were elevated to levels comparable to those seen with LPS stimulation alone (Figures 4B–D). This outcome was further substantiated by the expression of BIP protein, a critical marker of ER stress (Figure 4E), and an elevation of the proinflammatory marker IL-1β in the media, with no significant impact observed in C20:5 (n-3)-treated cells under PPARα inhibition (Figure 4F). Additionally, both functional assays for ROS production (Figure 4G) and mitochondrial depolarization (Figures 4H and I) showed no significant protection in C20:5 (n-3)-treated cells under PPARα inhibition, as the differences between C20:5 (n-3)-treated and untreated groups were not statistically significant. These observations indicate that the protective effects of C20:5 (n-3) were nullified by PPARα inhibition.

Collectively, these findings demonstrate that PPARα activation is essential for C20:5 (n-3) to exert its protective effects against LPS-induced oxidative and ER stress. The loss of C20:5 (n-3)'s beneficial effects in the presence of PPARα inhibition highlights the critical role of PPARα in modulating cellular responses to inflammatory and stress stimuli.

## 4. Discussion

Our study demonstrates that C20:5 (n-3) alleviates LPS-induced oxidative stress and inflammation through a mechanism involving the PPARα–NF-κB axis, with a pivotal role for FABP5 as a chaperone in facilitating C20:5 (n-3)'s binding to PPARα. These findings contribute to a growing body of research on the anti-inflammatory and cytoprotective effects of PUFAs in inflammatory and metabolic disorders, offering new insights into the precise molecular pathways involved.

The association between high-fat diets, increased circulating LPS levels, and chronic low-grade inflammation is well-established, with elevated LPS levels contributing to the development of obesity and insulin resistance through TLR4-mediated signaling pathways [8, 18]. LPS-induced inflammation not only promotes insulin resistance but also induces ER stress and oxidative stress, both of which play critical roles in the pathogenesis of metabolic diseases [19, 20]. ER stress arises from the accumulation of misfolded proteins, activating the UPR, which, when unresolved, can lead to apoptosis [21]. In our model, C20:5 (n-3) pretreatment significantly reduced the expression of ER stress markers, such as ATF4, DDIT3, and HSPA5, suggesting that C20:5 (n-3) can attenuate LPS-induced ER stress and restore cellular homeostasis. These findings align with previous studies highlighting omega-3 PUFAs' ability to reduce ER stress, which is known to contribute to the progression of obesity-related metabolic inflammation [22, 23].

Oxidative stress is another key factor exacerbating inflammation in metabolic disorders, as excessive ROS production can damage cellular components and activate inflammatory signaling pathways, including NF-κB [24]. In this study, C20:5 (n-3) significantly reduced ROS levels and preserved mitochondrial membrane potential, as shown by a stable aggregates-to-monomers ratio in the JC-1 assay, indicating that C20:5 (n-3) prevents mitochondrial dysfunction (Supporting Information 3: Figure S3), a major source of ROS production during inflammatory conditions [25]. Our findings align with the work of An et al. [26], who demonstrated that long-term omega-3 fatty acids supplementation mitigated tubulointerstitial injury in animals with chronic renal disease by reducing oxidative stress, inflammation, and fibrosis. Furthermore, we observed a reduction in HIF1α expression following C20:5 (n-3) treatment, which likely contributes to the decrease in ROS production. HIF1α is known to be upregulated during hypoxic and inflammatory conditions, promoting oxidative stress [27]. C20:5 (n-3)'s downregulation of HIF1α suggests a potential pathway by which it mitigates oxidative stress and maintains cellular integrity under inflammatory stress.

One interesting observation in our study is the essential role of PPARα and FABP5 in mediating C20:5 (n-3)'s protective effects. PPARα, a nuclear receptor involved in lipid metabolism and anti-inflammatory responses, has been previously associated with the beneficial effects of omega-3 fatty acids in metabolic health [28]. However, our study highlights a specific role for PPARα in modulating the inflammatory response to LPS, which has not been widely explored. We demonstrated that C20:5 (n-3) increases PPARα expression and requires its activation to exert anti-inflammatory and cytoprotective effects. This finding is supported by studies showing that PPARα activation is crucial in reducing ER stress and inflammation in obesity models [29]. The addition of FABP5 as a key mediator in this pathway provides further insight, in that FABP5 serves as a chaperone for long-chain fatty acids, facilitating their transport to PPARs [30]. The observed increase in FABP5 expression following C20:5 (n-3) treatment suggests that C20:5 (n-3) relies on this chaperone's function to effectively activate PPARα, thereby enhancing its anti-inflammatory properties.

Interestingly, our data show that C20:5 (n-3)'s effects on NF-κB are independent of the TLR4-IRF5 pathway. Although IRF5 is an essential downstream component of TLR4 signaling in the activation of NF-κB [31], C20:5 (n-3)'s reduction of NF-κB activation occurred without significant alteration in IRF5^+^ subsets. This suggests that C20:5 (n-3) might inhibit NF-κB activation through a pathway distinct from canonical TLR4-IRF5 signaling. Recent studies have indicated that PPARα can directly interfere with NF-κB activity, offering an alternative route for C20:5 (n-3)'s anti-inflammatory effects [32]. Our findings contribute to this understanding by demonstrating that C20:5 (n-3)'s anti-inflammatory action can bypass TLR4 signaling, instead relying on FABP5/PPARα to inhibit NF-κB. Previous reports have shown that C20:5 (n-3) activates GPR120/FFAR4 and PPAR*γ*, resulting in anti-inflammatory responses in adipocytes and macrophages [33, 34]. However, our data suggest that in the context of acute LPS stimulation, C20:5 (n-3) exerts its protective effects through upregulation of PPARα and FABP5 rather than through these canonical pathways. Notably, C20:5 (n-3) did not induce FABP4 expression, which is typically regulated by PPAR*γ*, and its effect on inflammation was preserved independently of TLR4–IRF5 signaling. These results highlight a novel and potentially stimulus-specific pathway, wherein C20:5 (n-3) targets mitochondrial function and oxidative stress through the FABP5–PPARα–NF-κB axis. We propose that future studies directly comparing the contribution of PPAR*γ*, PPARα, and GPR120 under identical inflammatory stimuli would provide further mechanistic clarity and potentially enable targeted therapeutic modulation of these pathways.

While our findings demonstrate a clear reduction in inflammatory responses following C20:5 (n-3) treatment, we acknowledge the relevance of assessing macrophage polarization status. Although C20:5 (n-3) has been implicated in promoting M2-like phenotypes in primary macrophages, our use of PMA-differentiated THP-1 macrophages presents a known limitation in this context. These cells, despite undergoing a 3-day resting period post-differentiation, exhibit a baseline M1-like transcriptional profile and respond poorly to M2-polarizing stimuli such as IL-4 consistent with previous reports [35, 36]. As such, we did not include M2 marker analysis to avoid drawing misleading conclusions from a system that does not accurately Model M2 transition. Future studies employing primary human macrophages or monocyte-derived macrophages (MDMs) will be critical to validate whether C20:5 (n-3)'s anti-inflammatory effects extend to functional M1-to-M2 switching under more physiologically relevant conditions.

To further confirm the role of PPARα in C20:5 (n-3)'s protective effects, we employed GW9662, a selective PPARα antagonist, to inhibit PPARα activity. Under PPARα inhibition, C20:5 (n-3)'s effects on ER stress, oxidative stress, and cytokine production were significantly reduced, indicating that PPARα activation is indeed necessary for C20:5 (n-3)'s anti-inflammatory and cytoprotective actions. Additionally, the functional assays revealed that C20:5 (n-3)'s ability to reduce ROS production and maintain mitochondrial membrane potential was abolished with PPARα inhibition, further confirming the receptor's role in regulating mitochondrial and oxidative stress responses.

**Figure 5:**
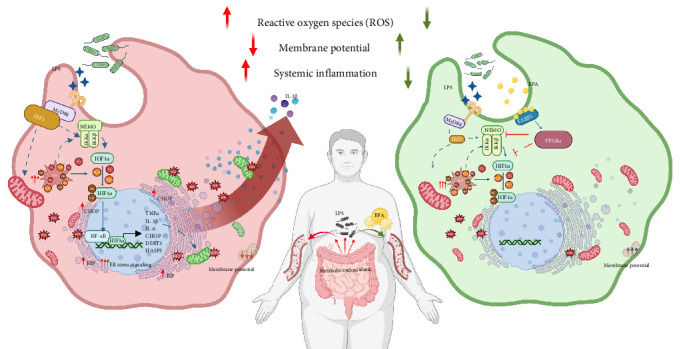
Schematic representation of the study findings. Metabolic endotoxemia as a key driver of systemic inflammation through the upregulation of LPS in circulation. The left panel illustrates the impact of LPS stimulation on macrophages, leading to increased production of reactive oxygen species (ROS), loss of mitochondrial membrane potential, and activation of inflammatory pathways mediated by HIF1α, NF-κB, and ER stress markers (CHOP, DDIT3, and HSPA5). This results in heightened systemic inflammation. The right panel demonstrates the protective effects of EPA treatment on LPS-stimulated macrophages. EPA enhances mitochondrial membrane potential, reduces ROS, and downregulates inflammatory markers, partly through the activation of FABP5/PPARα, which mitigates HIF1α, NF-κB, and ER stress signaling, ultimately alleviating systemic inflammation. This schematic underscores the therapeutic potential of EPA in reducing inflammation and oxidative stress associated with endotoxemia.

Overall, our study provides a mechanistic framework for C20:5 (n-3)'s protective effects in LPS-induced cellular stress, emphasizing the roles of PPARα and FABP5 in modulating the NF-κB pathway independently of TLR4-IRF5 signaling (Figure 5).

These findings suggest that targeting the FABP5/PPARα axis may offer a therapeutic strategy for mitigating inflammation and cellular stress linked to obesity and metabolic disorders. Given the demonstrated benefits of dietary EPA intake in reducing cardiovascular and metabolic risks, future studies should further investigate its therapeutic potential, alongside other PPARα agonists, in clinical models of metabolic endotoxemia. Exploring interactions between EPA and cellular stress pathways may also reveal novel approaches for managing chronic inflammation and oxidative stress in metabolic diseases.

## Figures and Tables

**Table 1:** List of qRT-PCR primers.

Reagent or resources	Source	Identifier
IL-1 b (human)	ThermoFisher	Hs01555410_m1
IL-6 (human)	ThermoFisher	Hs00174131_m1
TNF (human)	ThermoFisher	Hs01113624_g1
ATF4 (human)	ThermoFisher	Hs00909569_g1
DDIT3 (human)	ThermoFisher	Hs00358796_g1
HSPA5 (human)	ThermoFisher	Hs00607129_gH
PPARa (human)	ThermoFisher	Hs00947536_m1
PPARd (human)	ThermoFisher	Hs04187066_g1
PPARg (human)	ThermoFisher	Hs01115513_m1
HIF1a (human)	ThermoFisher	Hs00153153_m1
FABP4 (human)	ThermoFisher	Hs01086177_m1
FABP5 (human)	ThermoFisher	Hs02339439_g1
IRF4 (human)	ThermoFisher	Hs00180031_m1
IRF5 (human)	ThermoFisher	Hs00158114_m1
GAPDH (human) (VIC-Tamara)	Applied biosystems ThermoFisher	4310884E

**Table 2:** List of western blot primary antibodies.

Name (western blot)	Species	Company	Refrence/Catalog number
BiP (C50B12)	Rabbit mAb	Cell signaling technology	3177
CHOP (L63F7)	Mouse mAb	Cell signaling technology	2,895S
β-Actin (8H10D10)	Mouse mAb	Cell signaling technology	3,700S
PPARα (phospho s12)	Rabbit pAb	abcam	ab3484
FABP4 (D25B3) (XP)	Rabbit mAb	Cell signaling technology	3,544S
FABP5 (D1A7T)	Rabbit mAb	Cell signaling technology	39,926S

## Data Availability

The data supporting the findings of this study are available from the corresponding author upon reasonable request.

## Supporting Information

Supporting Information 1 The supporting Information includes the gating strategy for the flow cytometry data and raw Western blot immunoblot images, providing additional methodological details and unprocessed data to support the findings presented in the manuscript. Supporting Information 1. Figure S1: The gating strategy used for flow cytometric analysis in THP-1 cells is shown. Initially, a forward scatter (FSC) area versus FSC height plot was used to isolate single cells, thereby excluding doublets and cell aggregates. This “Singlets” gate, ensuring that only single-cell events were included in subsequent analysis. Next, a side scatter (SSC) versus FSC plot was applied to the singlet population to identify the THP-1 macrophage population based on cell size and granularity. Cells within this gate, labeled “THP-1 Cells,” represented 92%-98% of the singlet population, providing a highly purified subset of THP-1 macrophages for further examination. (A) To identify the proinflammatory profile, THP-1 cells were gated on a CD11b versus HLA-DR plot, selecting for CD11b+HLA-DR+ subsets. (B) For the isolation and identification of IRF5+ subsets, cells were gated on a CD11b versus IRF5 plot to identify the CD11b+IRF5+ population. This sequential gating approach ensures precise selection of specific cell subsets for downstream analysis.

Supporting Information 2 Figure S2: Vehicle controls (0.01% DMSO, 24% BSA, and media only) do not alter inflammatory, mitochondrial, or oxidative parameters in THP-1-derived macrophages. (A) IL-1β protein levels in cell culture supernatants assessed by ELISA reveal no significant changes across vehicle controls. (B) JC-1 assay quantifying mitochondrial membrane potential shows no significant difference in aggregate-to-monomer fluorescence ratio among 0.01% DMSO, 24% BSA, or media alone (C) Mean Fluorescence intensity of JC-1 monomers (MFI). (D) Intracellular ROS production measured by DCFH-DA flow cytometry also shows no difference among vehicle groups. Positive control (200 µM H_2_O_2_) induces a marked increase in ROS, validating the assay sensitivity.(E) Overlay histograms represent fluorescence distribution for DCFH-DA in the indicated groups. Data represent n = 3; ns = not significant (ANOVA).

Supporting Information 3 Figure S3: Mitochondrial membrane potential analysis by JC-1 staining in THP-1-derived macrophages reveals C20:5 (n-3)-mediated preservation under inflammatory stress. (A) Fluorescence microscopy images of THP-1-derived macrophages stained with JC-1 dye under various treatment conditions. JC-1 staining differentiates between healthy, polarized mitochondria (red fluorescence, JC-1 aggregates) and depolarized, dysfunctional mitochondria (green/yellow fluorescence, JC-1 monomers), serving as an indicator of mitochondrial membrane potential. Cells were pretreated with C20:5 (n-3) (200 μM), followed by stimulation with LPS (10 ng/mL). Red and green fluorescence channels are shown separately in the first two columns, with merged images in the third column. High-magnification insets (right panel) illustrate mitochondrial polarization status at the single-cell level. Scale bar = 200 μm (main panels), 20 μm (insets).
