# Supplementary material for: Eicosapentaenoic Acid (EPA) Alleviates LPS-Induced Oxidative Stress via the PPARα–NF-κB Axis
Source: Oxid Med Cell Longev. 2025 Jun 10;2025:3509596. doi: 10.1155/omcl/3509596 (PMC12173550; doi:10.1155/omcl/3509596)
Supplement: Supporting Information 1 — The supporting Information includes the gating strategy for the flow cytometry data and raw Western blot immunoblot images, providing additional methodological details and unprocessed data to support the findings presented in the manuscript. Supporting Information 1. Figure S1: The gating strategy used for flow cytometric analysis in THP-1 cells is shown. Initially, a forward scatter (FSC) area versus FSC height plot was used to isolate single cells, thereby excluding doublets and cell aggregates. This “Singlets” gate, ensuring that only single-cell events were included in subsequent analysis. Next, a side scatter (SSC) versus FSC plot was applied to the singlet population to identify the THP-1 macrophage population based on cell size and granularity. Cells within this gate, labeled “THP-1 Cells,” represented 92%-98% of the singlet population, providing a highly purified subset of THP-1 macrophages for further examination. (A) To identify the proinflammatory profile, THP-1 cells were gated on a CD11b versus HLA-DR plot, selecting for CD11b+HLA-DR+ subsets. (B) For the isolation and identification of IRF5+ subsets, cells were gated on a CD11b versus IRF5 plot to identify the CD11b+IRF5+ population. This sequential gating approach ensures precise selection of specific cell subsets for downstream analysis. [file 3509596.f1.pptx]

## Slide 1
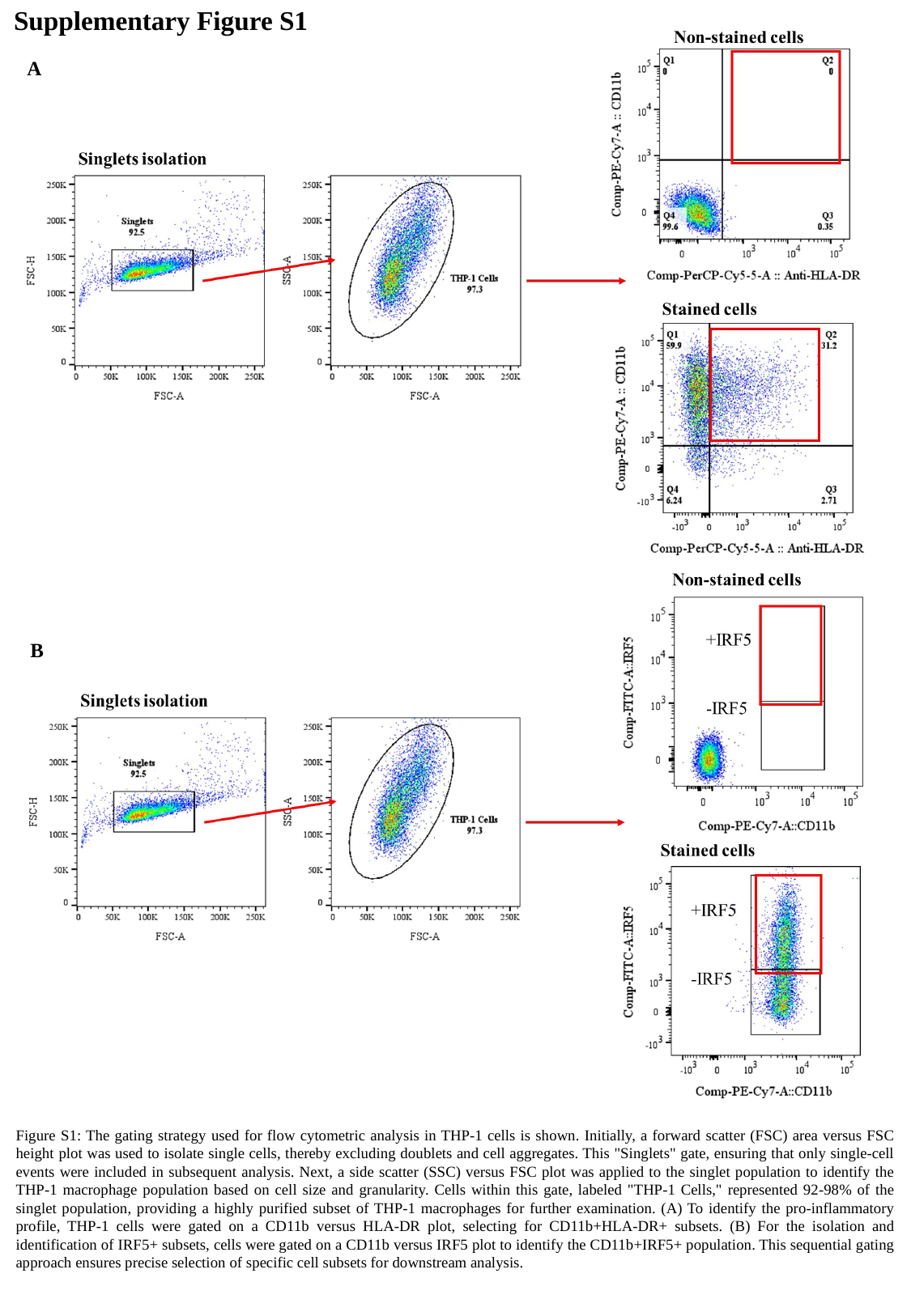

Supplementary Figure S1
A
B
Figure S1: The gating strategy used for flow cytometric analysis in THP-1 cells is shown. Initially, a forward scatter (FSC) area versus FSC height plot was used to isolate single cells, thereby excluding doublets and cell aggregates. This "Singlets" gate, ensuring that only single-cell events were included in subsequent analysis. Next, a side scatter (SSC) versus FSC plot was applied to the singlet population to identify the THP-1 macrophage population based on cell size and granularity. Cells within this gate, labeled "THP-1 Cells," represented 92-98% of the singlet population, providing a highly purified subset of THP-1 macrophages for further examination. (A) To identify the pro-inflammatory profile, THP-1 cells were gated on a CD11b versus HLA-DR plot, selecting for CD11b+HLA-DR+ subsets. (B) For the isolation and identification of IRF5+ subsets, cells were gated on a CD11b versus IRF5 plot to identify the CD11b+IRF5+ population. This sequential gating approach ensures precise selection of specific cell subsets for downstream analysis.
