# Supplementary material for: Eicosapentaenoic Acid (EPA) Alleviates LPS-Induced Oxidative Stress via the PPARα–NF-κB Axis
Source: Oxid Med Cell Longev. 2025 Jun 10;2025:3509596. doi: 10.1155/omcl/3509596 (PMC12173550; doi:10.1155/omcl/3509596)
Supplement: Supporting Information 2 — Figure S2: Vehicle controls (0.01% DMSO, 24% BSA, and media only) do not alter inflammatory, mitochondrial, or oxidative parameters in THP-1-derived macrophages. (A) IL-1β protein levels in cell culture supernatants assessed by ELISA reveal no significant changes across vehicle controls. (B) JC-1 assay quantifying mitochondrial membrane potential shows no significant difference in aggregate-to-monomer fluorescence ratio among 0.01% DMSO, 24% BSA, or media alone (C) Mean Fluorescence intensity of JC-1 monomers (MFI). (D) Intracellular ROS production measured by DCFH-DA flow cytometry also shows no difference among vehicle groups. Positive control (200 µM H2O2) induces a marked increase in ROS, validating the assay sensitivity.(E) Overlay histograms represent fluorescence distribution for DCFH-DA in the indicated groups. Data represent n = 3; ns = not significant (ANOVA). [file 3509596.f2.pptx]

## Slide 1
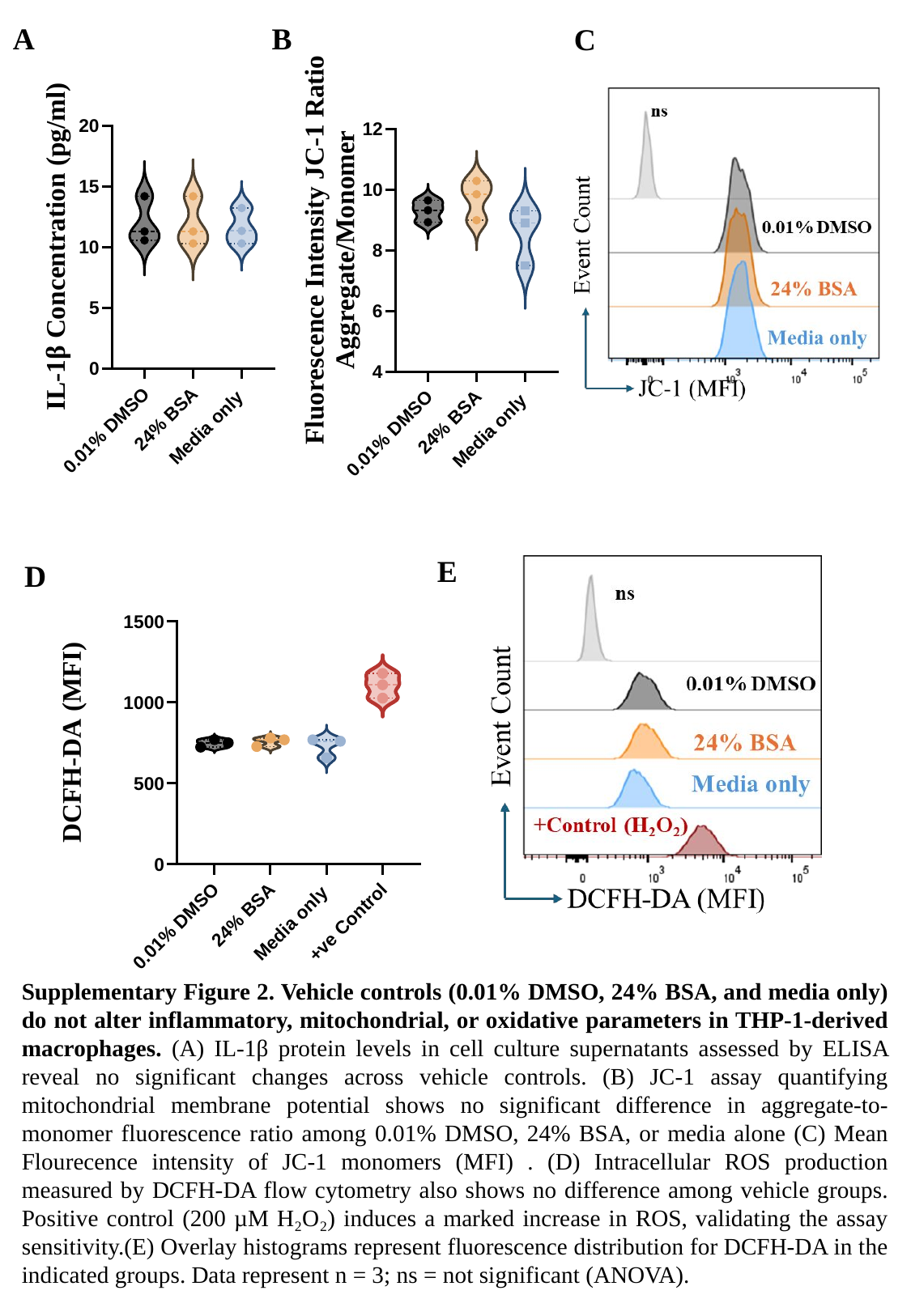

A
B
C
E
D
Supplementary Figure 2. Vehicle controls (0.01% DMSO, 24% BSA, and media only) do not alter inflammatory, mitochondrial, or oxidative parameters in THP-1-derived macrophages. (A) IL-1β protein levels in cell culture supernatants assessed by ELISA reveal no significant changes across vehicle controls. (B) JC-1 assay quantifying mitochondrial membrane potential shows no significant difference in aggregate-to-monomer fluorescence ratio among 0.01% DMSO, 24% BSA, or media alone (C) Mean Flourecence intensity of JC-1 monomers (MFI) . (D) Intracellular ROS production measured by DCFH-DA flow cytometry also shows no difference among vehicle groups. Positive control (200 µM H₂O₂) induces a marked increase in ROS, validating the assay sensitivity.(E) Overlay histograms represent fluorescence distribution for DCFH-DA in the indicated groups. Data represent n = 3; ns = not significant (ANOVA).
