# Supplementary material for: Eicosapentaenoic Acid (EPA) Alleviates LPS-Induced Oxidative Stress via the PPARα–NF-κB Axis
Source: Oxid Med Cell Longev. 2025 Jun 10;2025:3509596. doi: 10.1155/omcl/3509596 (PMC12173550; doi:10.1155/omcl/3509596)
Supplement: Supporting Information 3 — Figure S3: Mitochondrial membrane potential analysis by JC-1 staining in THP-1-derived macrophages reveals C20:5 (n-3)-mediated preservation under inflammatory stress. (A) Fluorescence microscopy images of THP-1-derived macrophages stained with JC-1 dye under various treatment conditions. JC-1 staining differentiates between healthy, polarized mitochondria (red fluorescence, JC-1 aggregates) and depolarized, dysfunctional mitochondria (green/yellow fluorescence, JC-1 monomers), serving as an indicator of mitochondrial membrane potential. Cells were pretreated with C20:5 (n-3) (200 μM), followed by stimulation with LPS (10 ng/mL). Red and green fluorescence channels are shown separately in the first two columns, with merged images in the third column. High-magnification insets (right panel) illustrate mitochondrial polarization status at the single-cell level. Scale bar = 200 μm (main panels), 20 μm (insets). [file 3509596.f3.pptx]

## Slide 1
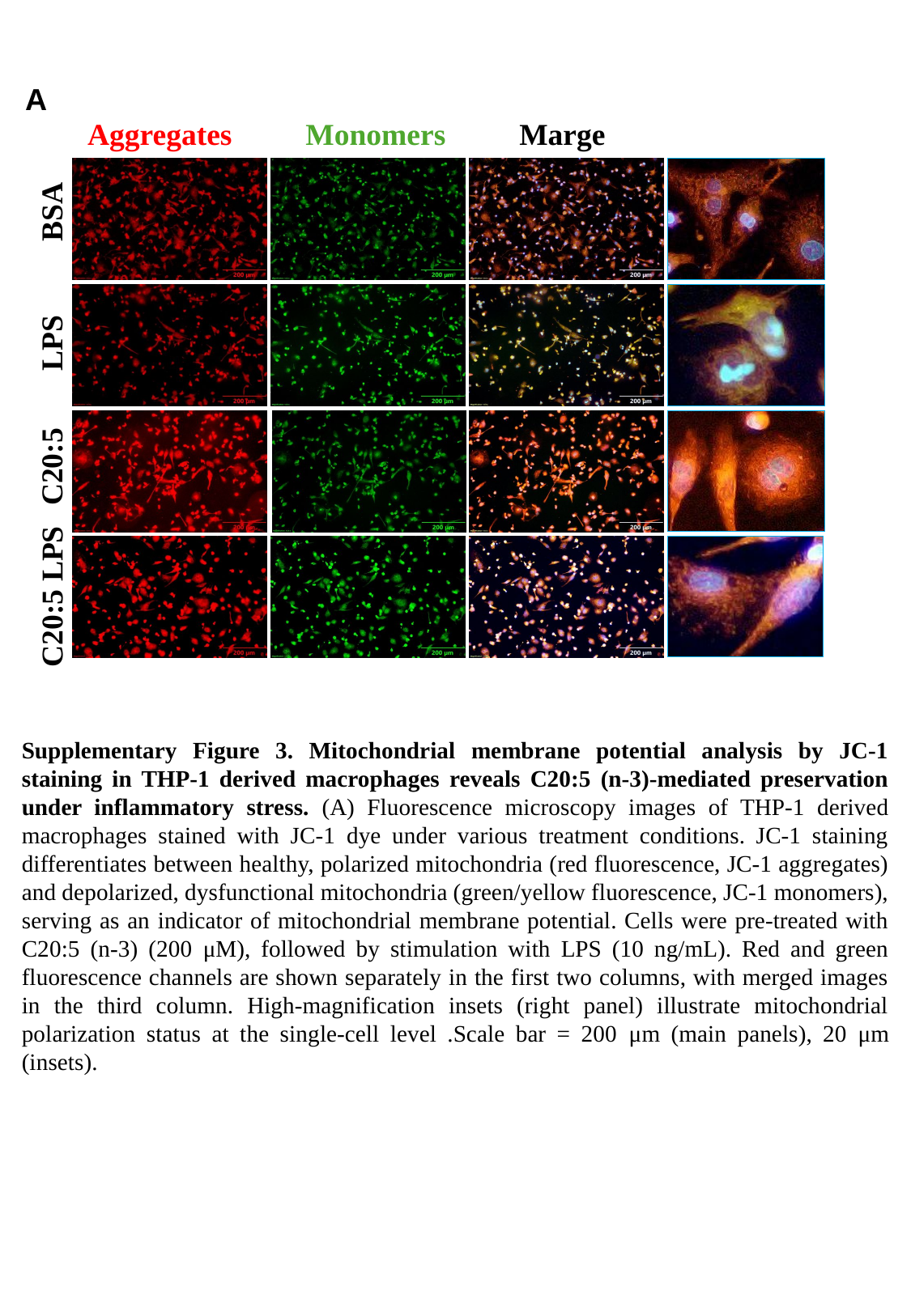

A
Aggregates
Monomers
Marge
BSA
LPS
C20:5
C20:5 LPS
Supplementary Figure 3. Mitochondrial membrane potential analysis by JC-1 staining in THP-1 derived macrophages reveals C20:5 (n-3)-mediated preservation under inflammatory stress. (A) Fluorescence microscopy images of THP-1 derived macrophages stained with JC-1 dye under various treatment conditions. JC-1 staining differentiates between healthy, polarized mitochondria (red fluorescence, JC-1 aggregates) and depolarized, dysfunctional mitochondria (green/yellow fluorescence, JC-1 monomers), serving as an indicator of mitochondrial membrane potential. Cells were pre-treated with C20:5 (n-3) (200 μM), followed by stimulation with LPS (10 ng/mL). Red and green fluorescence channels are shown separately in the first two columns, with merged images in the third column. High-magnification insets (right panel) illustrate mitochondrial polarization status at the single-cell level .Scale bar = 200 μm (main panels), 20 μm (insets).
